# Body–mind relaxation meditation modulates the thalamocortical functional connectivity in major depressive disorder: a preliminary resting-state fMRI study

**DOI:** 10.1038/s41398-021-01637-8

**Published:** 2021-10-23

**Authors:** Fangfang Chen, Xueyu Lv, Jiliang Fang, Tao Li, Jinping Xu, Xiaoling Wang, Yang Hong, Lan Hong, Jian Wang, Weidong Wang, Chao Wang

**Affiliations:** 1 College of Mathematics and Statistics, Shenzhen University, Shenzhen, 518060 China; 2 Guang’an Men Hospital, China Academy of Chinese Medical Sciences, Beijing, 100053 China; 3 Institute of Biomedical and Health Engineering, Shenzhen Institutes of Advanced Technology, Chinese Academy of Sciences, Shenzhen, 518055 China; 4 School of Psychology, Shenzhen University, Shenzhen, 518060 China; 5 Shenzhen Key Laboratory of Affective and Social Cognitive Science, Shenzhen University, Shenzhen, 518060 China

**Keywords:** Human behaviour, Depression

## Abstract

Mindfulness-based interventions such as meditation have increasingly been utilized for the treatment of psychological disorders and have been shown to be effective in the treatment of depression and relapse prevention. However, it remains largely unclear the neural mechanism of the therapeutic effects of meditation among depressed individuals. In this study, we investigated how body–mind relaxation meditation (BMRM) can modulate the thalamocortical functional connectivity (FC) in major depressive disorder patients and healthy controls. In the present study, we recruited 21 medication-naive adolescents with major depressive disorder (MDDs) and 24 matched healthy controls (HCs). We designed an audio recording to induce body–mind relaxation meditation. Resting-state fMRI (rs-fMRI) scans were collected before and after the BMRM intervention in both groups. The thalamus subregions were defined according to the Human Brainnetome Atlas, and functional connectivity (FC) was measured and compared to find brain regions that were affected by the BMRM intervention. Before the BMRM intervention, MDDs showed reduced FC of the bilateral precuneus/post cingulate cortex with the left posterior parietal thalamus and left caudal temporal thalamus, as well as an increased FC of the left occipital thalamus with the left medial frontal cortex. Moreover, aberrant FCs in MDDs at baseline were normalized following the BMRM intervention. After the BMRM intervention, both MDDs and HCs showed decreased FC between the left rostral temporal thalamus and the left inferior occipital. Given the small sample used in this study, future studies are warranted to evaluate the generalizability of these findings. Our findings suggest that BMRM is associated with changes in thalamocortical functional connectivity in MDDs. BMRM may act by strengthening connections between the thalamus and the default mode network, which are involved in a variety of high-level functioning, such as attention and self-related processes.

## Introduction

A major characteristic of major depressive disorder (MDD) is the persistence of sad mood and loss of interest or pleasure in doing normal activities for a prolonged period of time. In severe cases, MDD can lead to suicidal thoughts and behaviors [1, 2]. Mindfulness meditation is a contemplative practice that facilitates an increased engagement with the present moment and acceptance of bodily awareness. Heightened attention has been devoted to Mindfulness-based interventions (MBIs) in improving psychological wellbeing, and in particular, reducing depressive symptoms. For instance, previous studies have demonstrated the usefulness of MBIs in alleviating depressive symptoms and reducing the relapse rate for patients whose depressive episode are in remission [3, 4]. Furthermore, some studies have demonstrated the effectiveness of brief meditation interventions in improving mood, cognition, and self-regulation [5–7].

Body–mind relaxation meditation (BMRM) is a type of mindfulness training that has been implemented and utilized in many studies. BMRM allows for a high awareness of bodily sensations, engagement with breathing patterns, as well as stronger attention paid to both internal and external stimuli [8, 9]. BMRM has been demonstrated to be suitable for beginners and MDD patients, who may have difficulty suddenly entering into and remaining in a state of mindfulness.

Though there is burgeoning awareness of the benefits of mindfulness training for MDD patients, it still remains largely unclear the underlying mechanisms of how it works. fMRI functional connectivity, which allows for the identification of functional networks in the brain may provide insights into the underlying mechanism of mindfulness training in MDD patients. It has been used to examine the brain state-dependent activity and is well suited for studying meditation. For this study, we collected rs-fMRI data before and after a body–mind relaxation meditation induction session to examine the underlying mechanism of mindfulness training in MDD patients. We hypothesized that after a single brief session of guided meditation, participants would demonstrate functional changes in the brain.

The thalamus is one of the key structures in the brain that is changed by long-term meditation and plays an important role in awareness, alertness, attention, and emotional response to sensory experiences. Moreover, the thalamus has been referred to as a relay station for the processing of all sensory signals from various parts of the body to the cerebral cortex and then translating the information as it passes. Given its importance, even the smallest damage to the thalamus can negatively affect other brain areas. In a study by Eileen Luders, there was a marked increase of gray matter in the thalamus among individuals who engaged in long-term meditation compared to control subjects. This increase in the gray matter volume in the thalamus can be attributed to the meditators’ increased sense of awareness and focus during meditation [10]. In line with this finding, research has indicated that larger gray matter in the thalamus can increase positive emotions, longer-lasting emotional stability, and heightened focus in daily life [11].

Findings from numerous studies have revealed that the thalamus plays a far more complex role in cognitive functioning ranging from decision-making and attentiveness [12]. Furthermore, research has indicated that a dysfunction of the thalamus plays a critical role in the pathophysiology of MDD. For instance, depressed patients show reduced gray matter volumes in the thalamus [13]. In addition, a meta-analysis showed that hyperactivity of the thalamus may contribute to individuals with depression being highly sensitive to emotional stimuli [14]. Similar findings illustrate thalamic neurons are significantly higher in depression patients compared to healthy controls [15, 16] and thalamic lesions can induce depression in stroke patients [17, 18]. Furthermore, numerous structural and functional neuroimaging studies have shown deficits in the prefrontal–thalamo–limbic and limbic–striatal–pallidal–thalamic circuits in MDD patients [19]. Resting-state fMRI findings have shown abnormal thalamocortical functional connectivity in MDD patients [20, 21]. Kong and colleagues indicated a reduced parietal ROI-to-thalamus connectivity trend in MDD [22]. Liu and colleagues also found that the connectivity between the right posterior cingulate gyrus and right thalamus were negatively correlated with depression scores in unipolar depression [23].

Additional findings illustrate that the therapeutic effects of anti-depressants may be achieved by the regulation of the thalamocortical circuit FC. For instance, Salomons and colleagues illustrated that thalamocortical FC predicted clinical response to repetitive transcranial magnetic stimulation (rTMS) in MDD patients using resting-state (rs) fMRI [24]. Also when receiving negative valanced emotional stimulus, antidepressant medication such as selective serotonin reuptake inhibitors (SSRIs) inhibit the enhanced activity of the amygdala, thalamus, and other limbic regions in MDD patients [25, 26]. Electroconvulsive therapy can modulate the resting-state functional connectivity in the mediodorsal thalamus, which was associated with changes in depressive symptoms [27]. After treating MDD patients with the antidepressant escitalopram, increased long-range FCs were illustrated in the bilateral posterior cingulate cortex/precuneus and reductions in the right thalamus were observed in MDD patients [28]. Furthermore, the network dynamics of thalamocortical circuits have been proposed to be a promising pathway for brain stimulation treatment for MDD patients [22, 29]. These findings demonstrate the critical role that the thalamus plays in MDD.

To date, most studies have mainly focused on the neuroanatomical distinctions of MDD patients and healthy controls, and thus primarily examined the entire thalamus as a region of interest (ROI) to identify thalamic connectivity abnormalities in MDD. Nonetheless, human and animal studies have well-established that the thalamus serves a complex and multifarious brain region that organizes in many nuclei subserving diverse functions. Thus, it is inadequate to study the thalamus as an entire homogeneous structure with a unitary connectivity profile to examine its precise locations of thalamocortical connectivity associated with MDD neuropathology and treatment response.

In the current study, we aimed to determine whether specific thalamocortical networks were related to MDD and explore how BMRM experience can modulate the thalamocortical functional connectivity (FC) in MDD patients and healthy controls. We used rs-fMRI data to investigate the thalamocortical connectivity with separate thalamus subregions as seed ROIs. Based on previous reports, we hypothesized that the BMRM intervention will affect people with MDD and healthy controls differently.

## Materials and methods

### Subjects

Participants were 45 adults (21 MDD patients and 24 Healthy Controls) of Han Chinese ancestry. For both groups, participants were right-handed, between the ages of 18 and 50, and reported no contradiction to undergo an MRI scan according to a screening questionnaire. Recruited participants had to meet the following criteria for the MDD group: (1) SCID-IV diagnostic criteria for depression; (2) Hamilton Rating Scales for Depression (HDRS) score >20; (3) medication naive or withdrawn for 2 weeks before the rs-fMRI scanning and no other drug therapy; (4) no diagnoses of other psychiatric illnesses or severe physical illness or disease course longer than 2 weeks; (5) no history of qigong practice, yoga practice, or relaxation training. Furthermore, the criteria for the healthy controls were as follows: (1) no psychiatric disorders or severe physical disorders and (2) no history of qigong practice, yoga practice, or relaxation training.

The study’s protocol was approved by the Institute of Medicine Review Board at Guang’anmen Hospital of China Academy of Chinese Medical Sciences. And written informed consents were obtained from each participants. Before and after the BMRM induction section, both groups were scanned and rs-fMRI data was collected. For this study, we utilized an adapted version of BMBR in the experiment. The BMRM induction featured background music and a female broadcaster reading relaxing-inducing passages in standard Chinese Mandarin. Saishangqu, a type of soft and slow Chinese lute music, was utilized as the background music. The induction passage included two main sessions: (1) a whole-body scanning session and (2) a mind relaxation session. During the whole-body scanning session, participants were instructed to bring awareness and pay attention to every part of the body and focus their attention on their body’s physical sensations. During the mind relaxation session, participants were instructed to relax with statements such as “*now calm your mind… remove all your worries* …”. Participants listened to the BMRM induction for a total of 15 m and the body scanning session lasted for ~9 min [30].

### fMRI data acquisition

Before undergoing scanning, participants were instructed to keep their eyes closed, remain awake, not to think about anything specific, and to keep their heads still while in the MRI scan. MRI data were acquired on a 1.5 T GE Signal scanner using a standard GE whole-head coil. Blood oxygen level-dependent (BOLD) signals during functional runs were obtained by means of a T2*-weighted single-shot gradient echo-planar-imaging (EPI) sequence with the following parameters: TR/TE = 2500/30 ms; flip angle = 90°; data matrix = 64 × 64; FOV = 240 × 240 mm^2^; slice thickness = 3 mm with inner-slice gap = 0.5 mm; the sequence duration was 370 s for each subject, 150 time points were acquired.

### fMRI data preprocessing

All preprocessing was performed using DPARSFA 2.3 (Data Processing Assistant for Resting-State fMRI Advanced Edition, http://www.restfmri.net/forum/DPARSF) [31], which is based on statistical parametric mapping (SPM12, http://www.fil.ion.ucl.ac.uk/spm). The first 10 volumes were discarded for data equilibration. The remaining images were slice-time corrected and then realigned for head-motion correction. Subjects whose head motion exceeded 2 mm in any direction or rotation exceeded 2° were excluded from the study. Subsequentially, functional images were spatially normalized to the Montreal Neurological Institute (MNI) EPI template, resampled to a voxel size of 3 × 3 × 3 mm^3^, and then smoothed (Gaussian kernel full-width half-maximum, 6 mm). The time series for the whole brain were further preprocessed as follows: (1) six head-motion parameters, the averaged signals from the CSF and white matter, and the global brain signal were regressed; (2) to reduce the effects of low-frequency drifts and high-frequency noise, the time series were band filtered (0.01–0.08 Hz) and linearly detrended.

### Seed-ROI functional connectivity

**Fig. 1:**
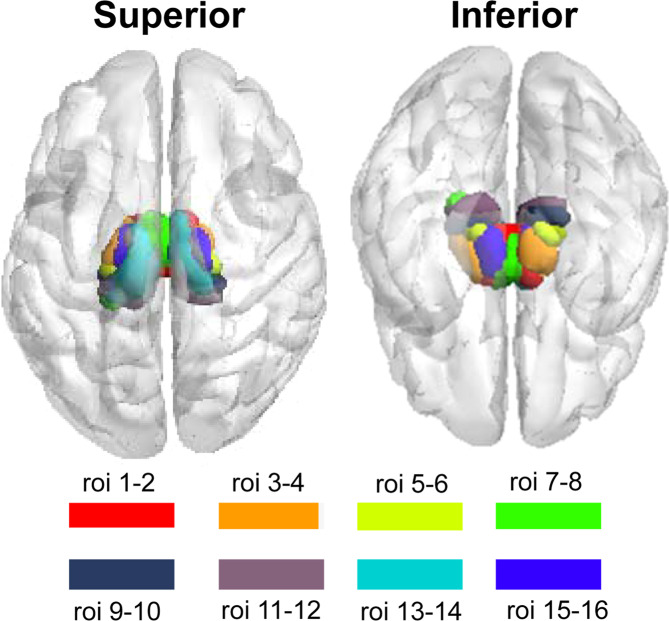
The anatomical location of thalamus subregions. The thalamus subregions containing the medial prefrontal thalamus (mPFtha, roi1, and roi2), the pre-motor thalamus (mPMtha, roi3, and roi4), the sensory thalamus (Stha, roi5, and roi6), the rostral temporal thalamus (rTtha, roi7, and roi8), the posterior parietal thalamus (PPtha, roi9, and roi10), the occipital thalamus (Otha, roi11, and roi12), the caudal temporal thalamus (cTtha, roi13, and roi14), and the lateral pre-frontal thalamus (IPFtha, roi15 and roi16).

The bilateral thalamus subregions were defined according to the Human Brainnetome Atlas. The Brainnetome atlas is an in vivo map based on fMRI and dMRI, with more fine-grained functional brain subregions and detailed anatomical and functional connection patterns for each area [32]. In this atlas, the thalamus was subdivided into eight subregions in each hemisphere (Fig. 1). The thalamus subregions contain the medial prefrontal thalamus (mPFtha, roi1, and roi2), the pre-motor thalamus (mPMtha, roi3, and roi4), the sensory thalamus (Stha, roi5, and roi6), the rostral temporal thalamus (rTtha, roi7, and roi8), the posterior parietal thalamus (PPtha, roi9, and roi10), the occipital thalamus (Otha, roi11, and roi12), the caudal temporal thalamus (cTtha, roi13, and roi14), and the lateral prefrontal thalamus (IPFtha, roi15, and roi16).

Each of the left and right thalamus subregions (roi1–16 in Fig. 1) was defined as seed ROIs. Functional connectivity maps were produced by computing Pearson correlation coefficients between the mean time series of each seed region and all the other voxels within a gray matter mask. Then, a fisher r-to-z transformation was applied to convert the correlation coefficient to *z* values to improve normality. Finally, we obtained z-FC maps of each subject for group statistics.

### Statistical analysis

Statistical analyses were performed using SPSS statistics 20.0 (SPSS Inc, Chicago, IL), SPM12 (http://www.fil.ion.ucl.ac.uk/spm), and MATLAB R2016a (The Mathworks, Natick, MA). There were four groups of data for statistical analysis: (1) the MDD patients’ FC maps before the BMRM (denoted as MDD_01), (2) the MDD patients’ FC maps after the BMRM (denoted as MDD_02), (3) the healthy controls’ FC maps before the BMRM (denoted as HC_01), and (4) the healthy controls’ FC maps after the BMRM (denoted as HC_02). A two-sample *t*-test (two-sided) was performed on the FC maps at baseline to find aberrant FCs in MDDs before the BMRM intervention and a partial correlation analysis was carried out to calculate association values between the HAMD scores and the aberrant FCs, while controlling for age and gender. To further explore the effect of BMRM on MDDs and HCs, a two-way mixed design repeated ANOVA with a between-subjects factor of diagnosis and a within-subjects factor of BMRM was performed using SPM software to identify the FCs with the main effect of diagnosis, a main effect of BMRM, and an interaction effect of diagnosis × BMRM. In the current study, the cluster-extent-based threshold was calculated using the Gaussian random field (GRF) method implemented in SPM. The resultant maps were corrected by GRF with voxel *p* < 0.001 and cluster *p* < 0.05 within a gray matter mask (cluster size >1215 mm^3^). For the FCs showing significant BMRM and interaction effect, post hoc paired *t*-tests and two-sample *t*-tests were applied to find how BMRM can affect FC patterns.

## Results

### Demographics and clinical characteristics

The demographics and clinical characteristics are presented in Table 1. The final groups (24 HCs, 21 MDDs) did not significantly differ in age (two-sample *t*-test: *p* = 0.74) or gender (Pearson chi-square *t*-test: *p* = 0.81).

**Table 1:** Group demographics and clinical measures.

	MDD patients	Healthy controls	*p* value
Age, year (mean ± SD)	36.05 ± 9.18	35.21 ± 7.92	0.74^a^
HAMD (mean ± SD)	30.48 ± 5.86	2.00 ± 2.02	<0.001^a^
Gender (female/male)	16/5	19/5	0.811^b^

*SD* standard deviation, *HAMD* Hamilton Depression Rating Scale.

^a^Indicates *p* values for two-sample *t*-test.

^b^Indicates *p* values for Pearson chi^2^ *t*-test.

### Seed-based resting-state functional connectivity

**Fig. 2:**
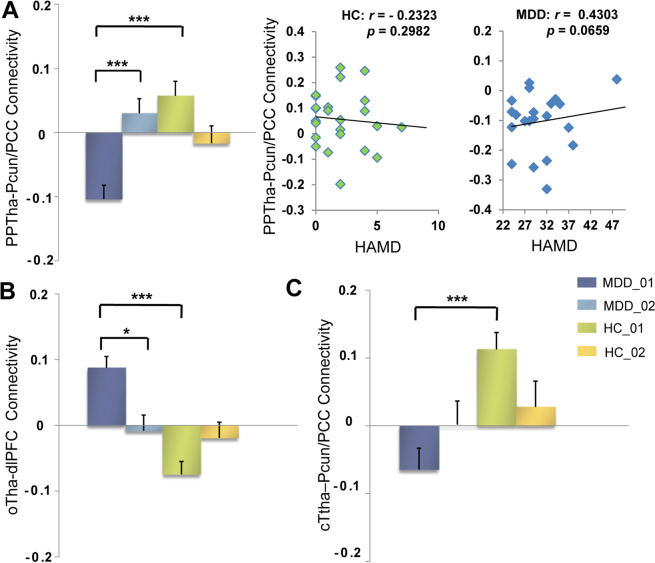
Functional connectivity showing significant differences in MDDs and HCs before BMRM. **A** Functional connectivity between the left posterior parietal thalamus (PPTha) and the bilateral precuneus/post cingulate cortex (Pcun/PCC) and its correlation with HAMD in HCs and MDDs. **B** Functional connectivity between the left occipital thalamus (oTha) and the left dorsolateral prefrontal cortex (dlPFC). **C** Functional connectivity between the left caudal temporal thalamus (cTha) and the bilateral precuneus/post cingulate cortex. HAMD Hamilton Depression Rating Scale, HC healthy control group, MDD major depressive disorder. *0.01 ≤ *p* < 0.05, **0.001 ≤ *p* < 0.01, ***p < 0.001.

Before the BMRM, MDDs showed reduced FC between the left posterior parietal thalamus and the bilateral precuneus/post cingulate cortex, reduced FC between the left caudal temporal thalamus and the bilateral precuneus/post cingulate cortex, and increased FC between the left occipital thalamus and the left medial frontal cortex (Table 2). Partial correlation analysis showed that only the positive correlation between HAMD scores and the FC of the posterior parietal thalamus with the bilateral precuneus/post cingulate cortex in MDD was marginally significant (*r* = 0.43, *p* = 0.066), and it was markedly different from the corresponding correlation in HCs (HCs: *r* = −0.232, *p* = 0.298; Fisher *z* = 2.136, *p* = 0.034). The above aberrant FCs in MDDs at baseline were normalized following the BMRM intervention (Table 2 and Fig. 2).

**Table 2:** Functional connectivity showing significant differences in MDDs and HCs before BMRM.

Seed region	Brain regions	BA	MNI coordinates			Voxels	*T* value
			*X*	*Y*	*Z*		
Left PPtha	Bilateral precuneus/post cingulate cortex	7/31/23	−3	−66	30	89	−4.4524
Left Otha	Left dorsolateral prefrontal cortex	9/6	−45	3	42	54	5.1487
Left cTtha	Bilateral precuneus/post cingulate cortex	7/31	−3	−66	30	47	−4.7954

*BMRM* body–mind relaxation meditation, *PPtha* posterior parietal thalamus, *Otha* occipital thalamus, *cTtha* caudal temporal thalamus, *BA* Brodmann area, *MNI* Montreal Neurological Institute.

**Fig. 3:**
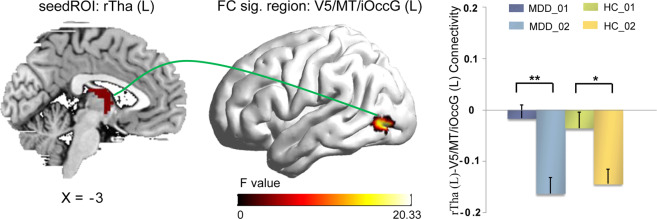
Main effect of BMRM. BMRM had the same effect on the healthy controls and MDD patients in the functional connectivity between the left rostral temporal thalamus (rTtha) and the left V5/MT+/inferior occipital gyrus (iOccG). Decreased FC values were observed after the BMRM in both groups. BMRM body-mind relaxation meditation, L left, R right. *0.01 ≤ *p* < 0.05, **0.001 ≤ *p* ≤ 0.01, ***p < 0.001.

BMRM had a significant main effect on the FC between the left rostral temporal thalamus (rTtha) and the left middle temporal visual cortex (MT/V5)/inferior occipital cortex (iOcc). Post hoc paired *t*-test found that after the BMRM intervention, the FC between those two regions became more negative in both groups, albeit much more significantly in the MDDs (Table 3 and Fig. 3).

**Table 3:** Functional connectivity showing significant differences in 2 groups (MDD/HC) × 2 conditions (before BMRM/after BMRM) repeated measure ANOVA.

Seed region	FC sig. region	BA	MNI coordinates			Voxels	*F* value
			*X*	*Y*	*Z*		
*Main effect of diagnosis*							
No significant FC							
*Main effect of BMRM*							
Left rTtha	Left V5/MT+/inferior occipital gyrus	37/19	−45	−78	−3	47	20.3317
*Interaction effect of diagnosis* *×* *BMRM*							
Left PPtha	Bilateral precuneus/post cingulate cortex	7/23	6	−54	27	78	21.4796

*BMRM* body–mind relaxation meditation, *ANOVA* analysis of variance, *FC* functional connectivity, *rTtha* rostral temporal thalamus x; *PPtha* posterior parietal thalamus, *BA* Brodmann area, *MNI* Montreal Neurological Institute.

**Fig. 4:**
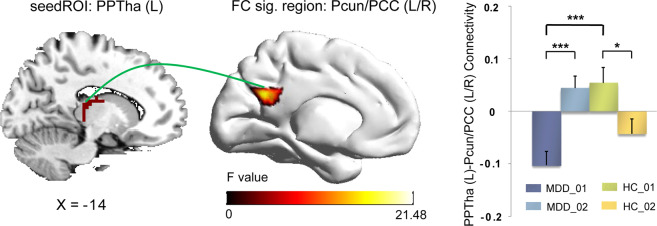
Interaction effect of Diagnosis × BMRM. BMRM had the different effect on the healthy controls and MDD patients in the functional connectivity between the left posterior parietal thalamus (PPTha) and the bilateral precuneus/posterior Cingulate Gyrus (PCun/PCC). After the BMRM, the MDD patients demonstrated significantly increased FC in left Pptha-bilateral PCun/PCC (turn from negative FC into positive FC), while the HCs demonstrated decreased FC in left PPTha-bilateral PCun/PCC. BMRM body–mind relaxation meditation, L left, R right. *0.01 ≤ *p* < 0.05, **0.001 ≤ *p* < 0.01, ***p < 0.00.

The FC between the left posterior parietal thalamus (PPtha) and the bilateral precuneus/posterior Cingulate Gyrus (PCC) showed an interaction of diagnosis × BMRM. At baseline, the MDD patients exhibited significantly decreased FC between those two regions compared with HCs. After the BMRM, a shift from negative to positive FC in left Pptha-bilateral precuneus/PCC was found in the MDDs, while decreased FC in left Pptha-bilateral precuneus/PCC was observed in the HCs (Table 3 and Fig. 4). After multiple corrections, we found no significant main effect of diagnosis.

## Discussion

In this paper, we examined the effects of BMRM intervention on thalamocortical FC among MDD patients and Healthy Controls. Our results showed a similar effect of BMRM on both MDDs and HCs and also a specific effect of BMRM on MDD patients.

Typically, the dorsal lateral prefrontal cortex (dlPFC) and thalamus are associated with attention control and cognitive flexibility [13, 33, 34]. Whereas the dlPFC helps to execute tasks that contribute to cognitive functioning, including working memory, attention, and decision-making [35], the thalamus is critical for enhancing and sustaining cortical connectivity. In recent years, numerous frameworks have begun to acknowledge the prominent role of the thalamus-frontal circuits in attention control and cognitive flexibility [34, 36–38]. Some studies support a negative attentional bias in depressed patients and mood states can influence depressed individuals’ attention and overall cognitive styles. Thus, the low mood observed in depression may contribute to attentional impairments and attentional impairments may in turn prolong low mood [39]. Previous studies have shown that the dlPFC was more functionally connected to the thalamus in MDD patients, suggesting a role of dlPFC-thalamus FC in the pathogenesis of MDD [40–42], which was consistent with our findings. Similarly, improved response to repetitive transracial magnetic stimulation in MDD patients was linked to the lower dlPFC-mediodorsal thalamus FC [24]. Furthermore, the mediodorsal thalamus has been linked to an array of cognitive functions such as working memory, behavioral flexibility, and goal-directed behavior [43]. Our findings of increased FC between the occipital thalamus and the dorsolateral prefrontal cortex in MDDs at baseline suggest negative attentional biases due to the persistent low mood prevalent among MDD patients.

The precuneus/PCC is widely recognized as the central core of the default mode network (DMN) [44], which plays a critical role in self-referential processing, self-consciousness, self-related mental representations, and first-person perspective taking [45–47]. The functional connectivity behaviors between the precuneus/PCC and other cortico-limbic regions is essential to determining how individuals perceive stimuli about themselves and process emotional events [48]. Although the thalamus is not considered to be a part of the DMN, the thalamus is both structurally and functionally connected to DMN regions and is considered to be crucial for DMN functioning [49, 50]. The thalamus receives information from the anterior cingulate cortex and the amygdala before transferring that information back to the cerebral cortex.

Depressive rumination is associated with abnormal FC in sgPFC-thalamus-precuneus/PCC circuit [51–53]. Other studies have reported that abnormal thalamus-PCC FC was related to stress and neuroticism. It is believed that individuals who are high in neuroticism are more vulnerable to stress and thus more susceptible to depression. Yin and co-workers demonstrated that the precuneus/PCC-thalamus FC is positively correlated with semantic memory and executive speed [54]. Previous studies have found reduced FC between the precuneus and the thalamus in MDD patients [23, 54, 55], which was consistent with our findings. Considering these previous findings, our study suggests that the disengagement of the posterior parietal thalamus and the caudal temporal from the DMN in MDDs at baseline may relate to the general memory bias prevalent in ruminative thinking, which largely contributes to the cognitive biases and impairments prevalent in MDD patients. Thus, reversal of these abnormalities by BMRM training suggests that modulating thalamus-DMN connectivity may serve as a potential therapeutic tool for the treatment of depressed individuals.

Furthermore, the precuneus and the thalamus have been consistently associated with an individual’s state of awareness [49, 56]. Although self-focus has been previously considered to be a single, monolithic state, it actually requires attention to either positive or negative self-aspects, of which focusing on the negative self-aspects may be associated with depression. Given that meditation training focuses on dealing with the present moment and increases attention to internal or external stimuli, our findings suggest that the negative rs-FC between the precuneus/PCC and the posterior parietal thalamus after the BMRM intervention in healthy controls may shift focus from relating sensory events to interoceptive awareness, which grounds participants to directly experience the present moment [57, 58].

New findings suggest that the anterior thalamic maybe involved in learning, episodic memory, as well as alertness modulation [59, 60]. Furthermore, the middle temporal visual cortex (MT or V5) is thought to be important in visual motion perception. On the contrary, evidence indicates that it may play a major role in attention control and imagery training [61, 62]. In conscious human subjects, electrical stimulation of cortical area MT is used to elicit reproducible illusory motion [63].

Zou and colleagues conducted a fMRI study with varying resting-state conditions (e.g., eyes open, eyes closed) to investigate the connection of the natural brain activity between the thalamus and the visual cortex. Their findings illustrated that in the eyes-closed condition, the whole thalamus showed a negative correlation with the visual cortex, which became less negative in eyes open condition [64]. Similarly, Hampson and colleagues found that there was a strong negative functional correlation between the V5/MT and the thalamus in the eyes-closed resting-state condition and the functional connectivity was significantly less negative when participants viewed continuous motion [65].

Other studies suggest that the thalamus and visual areas are crucial regions associated with electroencephalography (EEG) alpha power [66, 67]. For instance, by studying the relationship between the spontaneous variations of the alpha rhythm and the BOLD signals, researchers found that the alpha power was positively correlated with BOLD signal in the thalamus and negatively correlated with BOLD signal in the visual cortex [64, 68–71]. The alpha rhythm serves as a regulatory mechanism for information flow through the mind and is a sign of deep relaxation. It has been associated with alleviating stress, anxiety, discomfort, and pain [72]. The amount of Alpha waves is increased when our mind relaxes from any intentional or goal-oriented tasks. Some studies suggest that mindfulness meditation could regulate alpha rhythm in the cortex [73–75]. Although it is assumed that alpha rhythm originates from the occipital lobe, more recent papers have demonstrated that alpha rhythm may originate from the thalamus [76]. In our study, we found that the functional connectivity of rTtha-V5/MT was negative after the BMRM intervention in both groups, which suggests that body–mind relaxation meditation might play a role in the generation and modulation of the alpha rhythm in participants.

Nonetheless, some studies have indicated that the thalamo-visual cortex networks contribute to selective attention [77, 78], which allows individuals to focus and attend to relevant information in the environment and simultaneously tune out irrelevant information. Brain oscillations are important for sensory cognitive processes and integrate and process the entire brain with sensory information, and this information is sent to the thalamus, which consequentially allows for the experience of mindfulness. As a result, the practice of body–mind relaxation meditation may help individuals increase bodily awareness and recognition of its sensations, as well as improve interoceptive awareness skills [79, 80].

### Limitations

There were several limitations in our study. First, our sample was relatively small. Moreover, we investigated only the brain resting-state functional network connectivity. Ideally, studies that gather both behavioral and resting-state functional connectivity data from the same participants might be helpful to further validate our study’s findings. Furthermore, the parcellation scheme and the potential inter-individual variability of the thalamus subregions might also influence our results. Finally, the current findings suggest that modulating thalamus-DMN connectivity may be a potential therapeutic mechanism for body–mind relaxation meditation in MDD patients; however, this remains only a hypothesis. Further studies conducted with larger cohorts using multimodal data are warranted to further validate our results.

In conclusion, we investigated resting-state connectivity using functional magnetic resonance imaging before and after body–mind relaxation meditation in major depressive disorder patients and healthy controls. Our results illustrated that before the BMRM intervention, MDDs showed reduced FC between the left posterior parietal thalamus and the bilateral precuneus/post cingulate cortex, reduced FC between the left caudal temporal thalamus and the bilateral precuneus/post cingulate cortex, and increased FC between the left occipital thalamus and the left medial frontal cortex. In addition, we found that the functional connectivity between the left rostral temporal thalamus and the left middle temporal visual cortex (MT/V5) /inferior occipital cortex became more negative in both groups after the BMRM intervention; which has been indicated to be important in the generation and modulation of the EEG alpha rhythm. In addition, aberrant thalamo-precuneus/PCC functional connectivity in MDDs was normalized following the BMRM intervention, possibly implicating meditation-induced changes of the default mode network. Taken together, our results advance the knowledge of the influence of body–mind relaxation meditation in healthy individuals and those with major depressive disorder.
